# Emerging Nano-Formulations and Nanomedicines Applications for Ocular Drug Delivery

**DOI:** 10.3390/nano11010173

**Published:** 2021-01-12

**Authors:** Dawin Khiev, Zeinab A. Mohamed, Riddhi Vichare, Ryan Paulson, Sofia Bhatia, Subhra Mohapatra, Glenn P. Lobo, Mallika Valapala, Nagaraj Kerur, Christopher L. Passaglia, Shyam S. Mohapatra, Manas R. Biswal

**Affiliations:** 1 MSPN Graduate Programs, Taneja College of Pharmacy, University of South Florida, Tampa, FL 33612, USA; dawinkhiev@usf.edu (D.K.); zmohamed@usf.edu (Z.A.M.); vicharer@usf.edu (R.V.); smohapat@usf.edu (S.S.M.); 2 Department of Pharmaceutical Sciences, Taneja College of Pharmacy, University of South Florida, Tampa, FL 33612, USA; rjpaulso@usf.edu (R.P.); bhatias@usf.edu (S.B.); 3 Department of Molecular Medicine, Morsani College of Medicine, University of South Florida, Tampa, FL 33612, USA; smohapa2@usf.edu; 4 James A. Haley Veterans’ Hospital, Tampa, FL 33612, USA; 5 Department of Medicine, Medical University of South Carolina, Charleston, SC 29425, USA; lobo@musc.edu; 6 School of Optometry, Indiana University, Bloomington, IN 47401, USA; mvalapal@iu.edu; 7 Department of Ophthalmology, University of Virginia School of Medicine, Charlottesville, VA 22908, USA; nk8m@virginia.edu; 8 Department of Medical Engineering, College of Engineering, Tampa, FL 33620, USA; passaglia@usf.edu; 9 Department of Internal Medicine, Morsani College of Medicine, University of South Florida, Tampa, FL 33612, USA; 10 Department of Ophthalmology, Morsani College of Medicine, University of South Florida, Tampa, FL 33612, USA

**Keywords:** organic and inorganic nanoparticles, ophthalmic applications, clinical trials

## Abstract

Ocular diseases can deteriorate vision to the point of blindness and thus can have a major impact on the daily life of an individual. Conventional therapies are unable to provide absolute therapy for all ocular diseases due to the several limitations during drug delivery across the blood-retinal barrier, making it a major clinical challenge. With recent developments, the vast number of publications undergird the need for nanotechnology-based drug delivery systems in treating ocular diseases. The tool of nanotechnology provides several essential advantages, including sustained drug release and specific tissue targeting. Additionally, comprehensive in vitro and in vivo studies have suggested a better uptake of nanoparticles across ocular barriers. Nanoparticles can overcome the blood-retinal barrier and consequently increase ocular penetration and improve the bioavailability of the drug. In this review, we aim to summarize the development of organic and inorganic nanoparticles for ophthalmic applications. We highlight the potential nanoformulations in clinical trials as well as the products that have become a commercial reality.

## 1. Introduction

Vision stems from integrated and intricate communication between the eyes and brain. Visual impairment has a deteriorating effect on the life of an individual both physically and mentally. The global prevalence of ocular diseases based on 2015 statistics is an estimated 216.6 million people suffering from moderate to severe visual impairment and 188.5 million people affected by mild visual impairment. In the upcoming years, growth in the aging population will cause a spike in the number of cases of visual impairment, making it a major public health concern [1]. Common eye diseases include glaucoma, age-related macular degeneration (AMD), and diabetic retinopathy [2]. The delivery of therapeutics to the anterior segment of the eye is challenging, owing to the presence of anatomical and physiological barriers. Briefly, anatomical barriers are divided into static and dynamic barriers. Static barriers include corneal epithelium and blood-aqueous barrier, whereas dynamic barriers comprise tear drainage and conjunctival blood and lymph flow. On the other hand, physiological barriers include blinking action, tear film turnover, and nasolacrimal drainage. For drug delivery in the posterior segment, the major limitation is due to the presence of a blood-retinal barrier. Topical administration, like eye drops, is the most common and simple route for drug delivery to the anterior segment. However, the aforementioned problems result in poor bioavailability of topical applications, which has urged researchers to shift focus to novel nanotherapeutics for ophthalmic diseases [1,3]. Topical applications do not reach the posterior segment of the eyes, which makes intravitreal administration (IVT) of a drug the common route of administration. Although IVT injection efficiently delivers drugs to the posterior segment, it can be difficult to achieve for small and large bioorganic compounds like proteins and DNA, and some drugs need repeated injections to reach a therapeutic dose. Multiple injections can increase the risk of complications and possibly damage the eyes. Therefore, it is not a preferable option in treating ocular disease for most patients due to the lack of patient compliance [4].

In the past decade, clinical pharmacologists have focused on developing nanomedicine that can overcome barriers to provide a sustained and targeted release with minimal toxicity. The development of lipid-based nanocarriers has emerged into pharmaceutical industries and attracted attention from formulation scientists for its high drug solubility, substance release, and specific targeting. Organic nanoparticles, such as liposomes, polymeric nanoparticles, and dendrimers, are fabricated and developed to improve therapeutic drug delivery for the ocular disease [5,6]. Inorganic nanoparticles have garnered significant attention for their theranostic properties, substance release, and specific targeting. In this review, we aim to provide an insight into current treatment modalities based on nanotechnology for drug delivery in the eye. Finally, we will summarize the current nanoformulations under clinical trials and the potential formulations available on the market.

## 2. Nanotechnology in Ocular Diseases Therapy

Nanotechnology combines science and technology with the ability to manipulate structures and properties at the nanoscale size range, which lies between 1 and 100 nm. The ability to manipulate molecules on such a fine scale has application in many areas of basic science and in clinical treatment, diagnosis, and managing disease, which is known as “nanomedicine” [6]. Nanotechnology has been introduced since the 1980s in drug delivery systems to treat ocular disease [7]. The development of nanoformulation produces an ability to overcome ocular barriers, improve drug residence time on the cornea surface, increase permeability and bioavailability of the drug, reduce degradation of unstable drug and be well tolerated by the patient compared to the conventional drug [8]. Organic and inorganic nanoparticles (NPs) provide a new tool to address the unmet clinical needs, especially in the ophthalmic fields, with a significant improvement in drug delivery [5,6]. Therefore, various nanoparticle formulations have been fabricated, including lipid-based nanoparticles, nanosuspension, nanoemulsion, and metal-based nanoparticles.

## 3. Organic Nanoparticles

**Figure 1:**
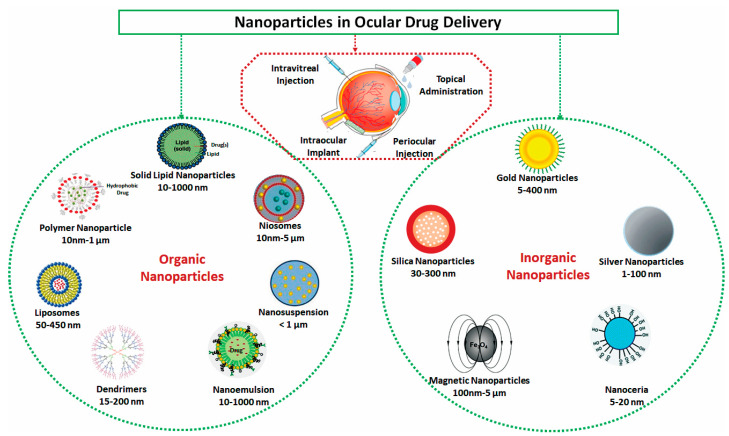
Schematic drawings for potential organic and inorganic nanoparticles for ophthalmic drug delivery.

The group of organic nanocarriers comprises liposomes, niosomes, dendrimers, solid-lipid NPs, polymers and protein/peptide-based NPs, which are fabricated from proteins, lipids, carbohydrates or other organic compounds (Figure 1). The use of organic nanoparticles seems to offer several advantages to facilitate drug or gene delivery to the ocular surfaces.

### 3.1. Liposomes

The liposome is a spherical-shaped nanoparticle with an aqueous core composed of one or more phospholipid bilayers, which resemble a cell membrane. Base on size and lamellarity, liposomes can be classified into three types: multilamellar vesicles, small unilamellar vesicles, and large unilamellar vesicles. Liposomes are widely used as nanocarriers in drug delivery systems and provide a significant advantage due to their size, biocompatibility, biodegradable, low toxicity, and potential to encapsulate hydrophilic, lipophilic, and amphiphilic drugs [9]. Due to their physicochemical and biophysical properties, liposomes can deliver drugs at different concentrations [10]. The liposome surface can be modified for targeted delivery, improving drug absorption to the posterior segment of the eyes, and the positive charge of the liposome can increase drug permeation to the cornea, prolonging drug release, which results in reducing multiple drug administration [7,10]. Law et al. [11] investigated acyclovir (ACV) embedded positively and negatively charged liposomes to target corneal penetration and absorption improvement. In an in vivo study, ACV-loaded liposomes were administered topically to the cornea surface, and the result suggested greater corneal permeation ability of ACV-loaded positively charged liposomes in comparison to negatively charged and plain ACV solution. The drug Edaravone loaded in submicron-sized liposomes was developed by Shimazaki et al. [12] for protection from light-induced retinal damage in mice. Their outcome demonstrated the eyedrop application of Edaravone encapsulated liposomes successfully inhibited light-induced reactive species oxygen production without any toxicity, making it a promising therapeutic potential in dry age-related macular degeneration management. Natarajan et al. [13] explored the therapeutic effect of liposome formulation to deliver latanoprost to treat glaucoma through subconjunctival injection administration. The liposomal formulation increased sustainability and drug targeting in the subconjunctival space. Along with this, latanoprost-encapsulated liposomes displayed an extended reduction of intraocular pressure (IOP) up to 50 days in rabbit eyes as compared to the conventional topical applications of eyedrops [13]. These promising effects have made liposomes an essential formulation in ophthalmic drug delivery. Bochot et al. [14] studied a novel approach to design an ocular drug delivery system that is based on the dispersion of PEGylated liposomes into a thermosensitive gel, which is made of a copolymer of ethylene oxide and propylene oxide (poloxamer 407). This approach offers new potentialities for ocular drug delivery.

### 3.2. Niosomes

Niosomes were first widely used in the cosmetic industry, but later their potential in drug delivery was studied for different routes of administration [15,16]. Niosomes are bilayer structured vesicles with a submicron size of 10 nm to over 5 μm composed of amphiphilic nonionic surfactants in the aqueous phase [7,17,18]. The essential properties of these vesicle nanocarriers offer the ability to entrap both lipophilic and hydrophilic drugs like liposomes but with the additional advantages of chemical stability, minimum toxicity due to its nonionic surface, and improved permeation of drug in the corneal cells [19]. The small size of niosomes offers an essential property to overcome the ocular drainage mechanism and produce better drug retention on the ocular surface [20]. Furthermore, compared to liposomes, niosomes are convenient for handling and storage and cost less to make this vesical more preferable to produce large-scale in the pharmaceutical industry [15]. Gugleva et al. [21] reported that there was a high encapsulation efficiency of doxycycline hyclate in niosomes prepared by sorbitan monostearate (span60) and cholesterol formulation, which produced a prolonged drug-release rate and was well tolerated in mice eye. Abdelbary and El-Gendy [22] achieved an encapsulation efficiency of gentamycin sulfate in niosomes that consisted of polyoxyethylene sorbitan monostearate, also called Tween-60 dicetyl phosphate (DCP), and cholesterol with an effect of controlled drug release from the ophthalmic delivery system comparable to the drug solution. Zeng et al. [23] prepared hyaluronic acid-coated niosomes for improving the ocular bioavailability of the tacrolimus drug. The combination of niosomes with hyaluronic acid coating on tacrolimus improved ocular adhesion increased precorneal drug retention, and enhanced permeation of the transcorneal area compared to noncoated niosomes.

### 3.3. Solid Lipid Nanoparticles

Recently, solid lipid nanoparticles (SLNs) have been explored as a novel delivery system that includes liposome nanoparticles as a colloidal lipid nanocarrier [24]. SLNs offer essential characteristics such as small size, large surface area, controlled drug release, excellent physical stability, and prevention of drug degradation. SLNs typically range from 50 to 1000 nm in size, are made of biodegradable, biocompatible materials, and are stabilized in an aqueous surfactant solution [25,26]. Recently, SLNs have attracted great attention as potential candidates for drug delivery to ocular surfaces due to their capability of increasing corneal absorption and thus improving bioavailability with sustained and controlled drug release properties for hydrophilic and lipophilic drugs [27,28]. A previous study incorporated cyclosporine-A into solid lipid nanoparticles for ocular delivery and showed that the nanoparticle achieved higher concentration in aqueous and vitreous humor due to the controlled and substance release effects of SNLs as compared to a cyclosporine-A solution [29]. Cavalli et al. [30] evaluated solid lipid nanoparticles for the delivery of the tobramycin (TOB) drug. The formulation was applied topically to New Zealand albino rabbits, and the results indicated prolonged preocular drug retention, which is associated with increased ocular bioavailability. Attama et al. [31] investigated the ocular permeation and sustained release effect of diclofenac sodium by synthesized SNL combined with phospholipid and a homolipid obtained from goat fat. It was found that drug-loaded with SNL produced sustained release, and permeation studies of diclofenac from SNL through human cornea construct in cell culture revealed the improvement of cornea permeation potential [31].

### 3.4. Polymeric Nanoparticles

Polymeric nanoparticles are spherical-shaped solid colloidal particles composed of biocompatible and biodegradable polymers in a size range from 10 nm to 1000 nm [32]. Some polymeric NPs that are obtained from natural polymers include chitosan, alginate, albumin, gelatin, and dextran, while some are obtained from synthetic sources such as polylactic acid (PLA), polyglycolide (PGA), poly(L-lysine), polyaspartic acid, poly-alkyl cyanoacrylate, and polyethyleneimine (PEI) which are biodegradable synthetic polymers. Based on preparation methods, polymeric nanoparticles can be fabricated into nanospheres and nanocapsules [25,33]. Nanosphere polymer has a polymeric matrix core structure, in which drugs can either be entrapped inside a matrix or attached to the polymer surface. Nanocapsules are formed with a liquid phase in the core, surrounded by a solid polymeric wall [6]. These polymer characteristics make them potential nanocarriers as it is possible to conjugate a drug molecule at the surface or encapsulate it inside and deliver it to a specific target site of action at a high concentration with low systemic toxicity [32]. Poly lactic-co-glycolic acid (PLGA) nanoparticles, a biodegradable copolymer, have been used in ophthalmic drug delivery due to their high potential to entrap hydrophilic/hydrophobic drug molecules with prolonged drug release [34]. Glycol chitosan cerium oxide nanoparticles have been studied in treating dry eye diseases in which cerium oxide was entrapped in glycol chitosan. These studies have shown the ability of the drug to enter the corneal cells and conjunctiva cells and promoting SOD2 expression [35]. Sharma et al. [36] found that chloro trimethyl-ammonium methyl methacrylate (Eudragit RS 100/RL 100) polymeric nanoparticles containing amikacin sulfate formulation produced high corneal adherence, which led to an increase in drug retention in the cornea, and drug controlled release efficiency was observed during in vivo studies. Another interesting research conducted by Li et al. [37] reported the efficiency of the combination of hydroxypropyl-beta-cyclodextrin (HP-β-CD) and PLGA (PLGA) nanoparticles for triamcinolone acetonide encapsulation in ophthalmic delivery. The in vitro drug release study demonstrated a higher rate of drug release from hydroxypropyl-beta-cyclodextrin (HP-β-CD) /PLGA nanoparticles compared to the suspension of drug-loaded PLGA without β-CD. Moreover, the significantly improved drug penetration of HP-β-CD /PLGA in the transcorneal area led to increased drug concentration in the aqueous humor, which makes an HP-β-CD/PLGA nanoparticle-loaded triamcinolone acetonide a potential formulation in eye drops [37].

### 3.5. Dendrimers

Dendrimers, a name derived from the Greek etymology meaning “tree”, are monodisperse macromolecules with well-defined size and a molecular structure composed of three major parts: (i) an inner core located inside the central molecule of the dendrimer, (ii) a highly branching unit forming a multilayer that links to the core called generation, and (iii) an outer surface with numerous valent functional groups [38]. Dendrimers have gained a prominent interest in the pharmaceutical application for drug delivery due to their tree-like branched structure with many covalent bonds allowing them to attach multiple functional groups, which can incorporate drugs inside dendrimers and conjugate them to the surface through covalent bonds. Their advantages include hydrophilic or lipophilic properties that offer control insolubility, and with their global shape, dendrimers can entrap small drug molecules inside their branches [39,40]. This unique architecture of dendrimers makes them an ideal nanocarrier for ophthalmic drug delivery with several purposes, such as improving permeability, water-solubility, biocompatibility, and bioavailability [41]. The most commonly used dendrimers-based nanocarriers are polyamidoamine (PAMAM) dendrimers that are hyperbranched [40]. It has been shown that polycationic PAMAM dendrimers are cytotoxic, and their toxicity increases as the generation increases, but hydroxy-terminated G4 PAMAM dendrimers are considered nontoxic due to their near-neutral surface charge that significantly reduces nonspecific retention and interactions in the tissues [42]. In other studies, PEGylation of PAMAM dendrimers has been shown to increase circulation time and biodistribution as well as reduce cytotoxicity of these dendrimers [43]. Mishra and Jain [44] have evaluated the intraocular pressure-lowering potential of acetazolamide (ACZ)-loaded poly-(propylene imine) dendrimer nanostructures for topical ocular administration. Acetazolamide-loaded dendrimer formulation was topically applied to adult male New Zealand albino rabbit eyes. The results showed that the drug-loaded dendrimer formulation caused a reduction in IOP for an extended time, approximately 4 h, compared to ACZ solution, which only showed a reduction effect up to 2 h [44]. In another study, PAMAM dendrimer was synthesized with primary amine and carboxylate surface groups to obtain better drug corneal permeation and properties during in vitro release. A dendrimer-based formulation was used for puerarin drug delivery and successfully tested on the albino rabbit model for ocular hypertension and cataracts glaucoma treatment [45]. Marano and coworkers [43] have designed lipophilic amino-acid dendrimer for anti-vascular endothelial growth factor (VEGF) oligonucleotide (ODN-1) retina delivery to treat choroidal neovascularization. It was reported that dendrimer conjugated ODN-1 significantly inhibits choroidal neovascularization (CNV) progression, and no side effects were observed during examination [46]. Given all the benefits of dendrimers, these formulations have the potential to improve the efficacy of drugs in ophthalmology.

### 3.6. Nanosuspensions

Poor solubility and low availability are the main concerns for the formulation of drugs in pharmaceutical industries as conventional formulations cannot overcome these problems, and over 40% of newly discovered drugs are reported to have low water solubility. Many strategies have been employed to improve poor drug solubility, but due to limitations in these strategies, scientists have found nanosuspensions to be a promising additional approach in drug delivery. Nanosuspensions are colloidal dispersions in nanoparticle size which have a surfactant as a stabilizer. Administration of nanosuspension offers benefits in sustained release of poorly soluble drug in the ocular surface, which leads to the increase of retention time [47,48,49]. Nanosuspensions have been reported in oral and topical formulations to enhance ocular drug bioavailability. In previous studies, a nanosuspension of ibuprofen sodium salt (IBU) sodium salt-coated chloro trimethyl-ammonium methyl methacrylate (Eudragit RS 100) polymer tested on rabbit eye by topical administration demonstrated penetration improvement to the anterior segment of the eye, extended drug release, and increased level of drug in the aqueous humor [50]. Some glucocorticoids used topically to treat eye inflammation, such as dexamethasone, hydrocortisone, and prednisolone, have been formulated in nanosuspension, showing an effect in prolonged drug absorption with higher drug bioavailability in ocular drug delivery, thus reducing frequent drug administration [51,52].

### 3.7. Nanoemulsion

Nanoemulsion is a colloidal system composed of two liquid phases, oil dispersed in water or water dispersed in oil, in which the nanoemulsion droplets with size 20–500 nm are stabilized by surfactants such as cetyltrimethylammonium bromide and sodium dodecyl sulfate [53,54]. The small size of droplets offers a high surface area with a significant potential to provide better efficacy in ophthalmic drug delivery with its benefits of improving ocular permeability and high bioavailability [55]. Nanoemulsion is considered to have low surface tension and greater drug spreading on the cornea, which can mix properly with the precorneal constituents. This enhances the drug contact time in the corneal epithelium [56]. The study by Ismail et al. [57] on antiglaucoma drug dorzolamide hydrochloride nanoemulsion showed promising effects on ocular therapy. The drug formulation offered prolonged effect with a quick onset of action and thus reduced frequent administration of eye drops. Tayel et al. [58] developed terbinafine hydrochloride-loaded nanoemulsion gels. The formulation that was developed indicated extended drug residence time and bioavailability improvement. Clinical research conducted by Mahboobian et al. [59] evaluated the uptake of acyclovir nanoemulsion in the bovine cornea following transcorneal permeation. The results showed increased cornea permeation of acyclovir through the corneal membrane cells, and no irritation occurred on the rabbit eye studied, which indicates that the formulation can be considered safe for treating ocular infection [59]. Table 1. Summarizes the applications of organic nanoparticles for drug delivery to ocular surfaces.

## 4. Inorganic Nanoparticles

Inorganic nanoparticles comprise mainly metallic nanoparticles (Figure 1) and quantum dots. Metallic nanoparticles have gained much attention over the past decade since Faraday proved that they could exist in solution [35]. They can be categorized into four different groups: metal nanoparticles, metal oxide nanoparticles, doped metal–metal/oxide–metal nanoparticles, and metal sulfide, and metal–organic frameworks. Metal NPs such as silver (Ag), gold (Au), copper (Cu), magnesium (Mg), titanium (Ti), platinum (Pt), zinc (Zn), and iron (Fe) nanoparticle have been investigated in different research areas and have succeeded as efficient, stable drug delivery platforms with fewer side effects and powerful imaging probes. Doped metallic nanoparticles, like ZnO doped with Co, were reported to have lower toxicities than single metals with better properties [60,61]. In the ocular drug and gene delivery field, metals such as cerium oxide (CeO2) NPs and gold NPs have demonstrated their potential antioxidant capabilities with a high safety profile after delivery to the eye and high chemical stability.

### 4.1. Gold NPs (AuNPs)

AuNPs are one of the noble metals that are known for their unique optical properties induced by the popular phenomenon of localized surface plasmon resonance (LSPR). This phenomenon is strongly influenced by the AuNP shape and is the main reason for its penetrative ability to the biological tissues [62]. AuNPs have other advantages that make them popular in the nanomedicine field, like their chemical stability, surface functionalization, biocompatibility, and unique surface and size characteristics [63]. The biocompatibility and internalization capability of different AuNP morphologies (rods, spheres, and cubes) have been assessed in retinal pigment epithelium (RPE) cells. Spheres and cubes of 50 and 100 nm showed no cytotoxicity and good internalization properties; however, rod-shaped particles were less biocompatible [64]. Dong et al. [65] showed that resveratrol coated AuNPs with a median size of 20 nm and doses of 200–300 mg/kg injected in streptozotocin-induced diabetic rats for 3 months could provide a protective effect against diabetic retinopathy. This protective effect of AuNPs could help to regain the balance of the stimulators and inhibitors of the angiogenesis process through the inhibitory effects of the ERK1/2 pathway and nuclear factor kappa B (NF-kb) expression, which could reduce inflammation and permeability of the blood-retinal barrier in the diabetic rats. Furthermore, there was a significant reduction of all the retinal mRNA expressions of vascular endothelial growth factor (VEGF-1), tumor necrosis factor-alpha (TNFα), and interleukin 6 (IL-6) [65]. In another study of the topical application of AuNPs, Pereira et al. [66] showed that AuNPs could decrease the intraocular oxidative damage and inflammation in endotoxin-induced uveitis rat models.

The treatment of choroid-retina endothelial (RF/6A) cells with AuNPs of 3–5 nm successfully inhibited the VEGF-induced RF/6A cell migration through the Akt/eNOS pathways. In their cell viability and cell adhesion studies, they found neither cytotoxic effects on AuNPS on RF/6A nor detrimental effects on the normal physiological adhesion of cells to fibronectin [62]. Hayashi et al. [67] examined the feasibility, biodistribution, and effects of the subretinal injections of immunoglobulin G (IgG) adsorbed on gold nanoparticles (GNPs) in rabbits’ eyes for three months and in ARPE-19 cultured cells, and they found that GNPs was successfully delivered into photoreceptor cells and RPE in rabbits and reported no cytotoxicity in ARPE-19 cells. Moreover, in inherited retinal dystrophies gene delivery therapies, AuNPs have been examined as a safer and more efficient alternative to the viral-based approaches in RPE cells. The preliminary results of Trigueros et al. [68] have shown that plasmid DNA-wrapped AuNPs with a size of 40 nm could be successfully internalized in differentiated ARPE-19 cells with good transfection efficiency. These results were supported by the early expression of a reporter gene that was noted at 16 h post-transfection. Their results show a potentially successful gene delivery route to RPE cells using AuNPs; nevertheless, they propose that the interaction mechanism of cell to AuNPs must be better understood to increase the transfection efficiency of these particles and escape the autophagic pathways of the particles to ensure sustained gene expression effect of their system [68]. Generally, AuNPs are considered as a core-shell system, at which gold is the inert inner core, and the drug can be conjugated to either its core or outer active layer through thiol linkages. As mentioned earlier, gold is biocompatible, and it is approved for internal medicine; however, the main challenge for AuNPs to be used as drug delivery vehicles in the clinical practice is their non-degradable gold core that elongates their excretion from the body [69]. Interestingly, the successful passage of AuNPs of the size 20 nm through the blood-retinal barrier (BRB) and their distribution in all retinal layers after their intravenous injection into C57B1/6 mice without showing any structural damage or increased cell death compared to cells without NPs by Kim et al. [70] raise their potential existence in future clinical trials.

### 4.2. Silver Nanoparticles (AgNPs)

Silver nanoparticles (AgNPs) have been used widely in the nanomedicine field due to their advantages of the unique chemical and physical characteristics, large surface area to volume ratio, low production cost, and biocompatibility that make them good candidates as drug delivery carriers [71]. Various physical and chemical methods are being used to synthesize and stabilize the AgNPs. Out of these, the chemical method is most common, which includes chemical reduction, electrochemical techniques, reducing agents, physiochemical reduction, and radiolysis [72]. AgNPs synthesis using these chemical methods is experiencing problems such as stability and agglomeration of NPs, shape, and size of particles. The synthesis of AgNPs requires (a) silver precursors, (b) reducing agents that include organic (e.g., ethylene glycol, ascorbic acid, glucose) and inorganic (e.g., hydrazine, sodium borohydride), (c) capping agents or stabilizing agents such as polyvinylpyrrolidone (PVP) [72]. Capping agents offer electrostatic and steric stabilization effects for AgNPs dispersion in suspensions [73].

AgNPs have been reported as effective nanoparticles in the treatment of cancer and infections due to their powerful tumor-killing and bactericidal effects. In the ocular field, AgNPs have shown successful delivery of therapeutic agents to eyes and have been used as a coating agent for contact lenses. In addition, AgNPs alone or in conjugation with natural plant extracts demonstrated significant anti-angiogenic, antioxidant, anti-glycation end products, and anti-cataractogenic effects in various cell culture systems and animal models for ocular diseases [74]. Anbukkarasi et al. [75] investigated the antioxidant and anti-cataractogenic effect of AgNPs with a size range of 15–50 nm formulated with the ethanolic extract of T. divaricate leaves, which reportedly have antioxidant and anti-cataractogenic pharmacological effects. The study designed an in vitro selenite-induced cataractogenic model to evaluate the effects of AgNPs against the dense opacification of Wistar rat lenses. The results suggested that the formulated AgNPs acted as a potent antioxidant by dose-dependent scavenging of DPPH and H_2_O_2_ free radicals, which are the main triggers of cataract formation [75]. Kalishwaralal et al. [76] tested the anti-angiogenic properties of formulated AgNPs in a size range of 40–50 nm, acting as an inhibitor of VEGF, which induces cell proliferation and migration in bovine retinal endothelial cells (BRECs). The findings of Gurunathan et al. [77] suggested that AgNPs could inhibit angiogenesis via the inhibition of the PI3K/Akt signaling pathways. Sheikpranbabu et al. [78] studied the effects of AgNPs with a size of 50 nm on advanced glycation end-products (AGEs)-induced endothelial cell permeability in porcine retinal endothelial cells (PRECs), and the studies demonstrated that AGE-induced permeability was inhibited by AgNPs through the Src kinase pathway.

Since the nineteenth century, silver nitrate has been known for its potential antimicrobial effects as it was widely used against ophthalmia neonatorum. Xu et al. [79] investigated the antifungal effects of polymeric AgNPs of sizes ranging from 20 to 30 nm against three different types of filamentous fungi isolates that cause fungal keratitis. The antifungal effects of AgNPs in the fungal cultures were compared to those of the antifungal natamycin, and the AgNPs demonstrated a significantly superior antifungal activity that was 4–32 times higher than natamycin.

Despite the advantages that AgNPs offers as antifungal, antioxidant, anti-angiogenic, and anti-inflammatory agents, their limited advancement in the ocular drug delivery field goes back to their reported toxicity in several studies. The widely reported toxicity is related to the release of silver ions from the nanoparticle surface that induces irritation to different ocular parts. Jun et al. [80] investigated the toxicity of silver NPs at a dose of 0.4 mg/L and reported downregulation of several lens crystalline genes by the possible cell death or the nuclear DNA or RNA export blockage. Kim et al. [81] also observed edema and conjunctival redness after a one-hour exposure of New Zealand white rabbits’ eyes to AgNPs. In a study, AgNPs with sizes of 22.4 nm and 42.5 nm, respectively, induced cytotoxic effects and activation of the reactive oxygen species (ROS) system in bovine retinal endothelial cells (BRECs) [82].

### 4.3. Cerium Oxide Nanoparticles (Nanoceria-CeO_2_-NPs)

Cerium is a rare earth element in the lanthanide series of the periodic table and is considered a potent antioxidant. The mechanism by which CeO_2_-NPs of 3–5 nm exhibits its catalytic antioxidant reaction involves two oxidation states, a reduction of Ce4+ to Ce3+ and a loss of an oxygen atom [83]. This reaction creates vacancies for reactive oxygen species (ROS) in cells to replace the loss of oxygen, which mimics the antioxidant enzymatic activities of superoxide dismutase (SOD) and catalase [84]. Nanoceria has proved their efficacy as antioxidants to provide protection in many diseases and act as neuroprotective, radioprotective, cardioprotective, as well as anti-inflammatory agents [85].

In the ophthalmic field, CeO_2_-NPs have been investigated in several animal models of age-related macular degeneration (AMD) and showed promising antioxidant, anti-inflammatory, and anti-angiogenic effects. Moreover, they were reported to provide long-term neuroprotection for photoreceptors in retinitis pigmentosa (RP) models without collateral adverse effects. They were also used as an effective anti-cataractogenic material and were reported to be safe on lens cells [86]. Nanoceria is mainly fabricated by simple wet chemistry methods, as explained by Karakoti et al. [87], and their PEGylation and liposomal encapsulation enable them to permeate the cornea [86]. Chen et al. [88] reported that fabricated CeO_2_-NPs of 20 nm particle size could prevent the increased concentration of free radicals in primary cell cultures of rat retinas. These findings prompted them to test the NPs in vivo, where a group of albino rats was exposed to 2700 lux of light for 6 h. The light exposure normally results in damage to 50–60% of photoreceptors, but their electroretinogram (ERG) data showed that a significant number of photoreceptor cells were rescued after posttreatment with CeO_2_-NPs [88]. Kong et al. [85] testing of nanoceria on P34 mice including ROS reduction, increase in the expression of neuroprotection associated genes, and inhibition of the apoptosis signaling pathways, all of which delayed the degenerative condition of photoreceptor cells.

In a study by Fiorani et al. [89], pretreatment of albino Sprague–Dawley rats with intravenous and intravitreal injections of CeO_2_-NPs for 3 weeks could prevent neuronal death induced by 1000 lux light for 24 h and helped preserve the integrity of the outer nuclear layer. The study also demonstrated that nanoceria remained stable in the outer layer and could inhibit the microglial activation and migration toward the outer nuclear layer for an extended time [89]. Intravitreal injections of nanoceria were also tested by Zhou et al. [90], who found inhibition of neovascularization in Vldlr knockout mice through their inhibitory effects of the VEGF cascade and their initiation of antioxidant genes expression pathways; they reported these results as promising findings that could make nanoceria good candidates in the treatment of AMD and diabetic retinopathy diseases. In a study by Bhargava et al. [91], nanoceria of 5–10 nm could increase the survival of rod and cone photoreceptors in culture by helping the maintenance of the cell line, which allows for improved drug screening experiments on these cell lines.

### 4.4. Mesoporous Silica Nanoparticles (MSNs)

Mesoporous silica nanoparticles (MSNs), one of the most well-studied inorganic nanoparticles for drug delivery. These are mesoporous systems made up of silica with particle sizes of 30–300 nm. They can adsorb exceptionally large quantities of drugs in their pores. Unique properties of MSNs include large specific surface area and pore volume, controllable particle size and better biocompatibility make them excellent nanoplatform for biomedical applications and drug delivery [92]. Park et al. [93] conducted a study to evaluate the cytotoxicity of three different sizes (50 nm, 100 nm and 150 nm) of silica nanoparticles (SiNPs) on ocular surface cells such as human corneal epithelial cells (HCECs). Experimental results suggested that cellular autophagy and mammalian target of rapamycin (mTOR) pathways activated with the addition of SiNPs without inducing any significant cytotoxicity in cultured HCECs.

An eye drop, brimonidine showed limited effectiveness in the treatment of glaucoma because of its rapid clearance from preocular space. To resolve this issue, a study was conducted by Kim et al. [94] to deliver brimonidine using amino-functionalized mesoporous silica (AMS) particles so that AMS particles form adhesion with the mucous layer and allow high preocular retention time. Because of the presence of mesopores, AMS encapsulated brimonidine released the drug in a sustained manner over 8 h. BMD-AMS stayed in preocular space for up to 12 h when topically administered into the eyes of a rabbit. To measure the in vivo efficacy, the variance in IOP and brimonidine concentration was examined after administering BMD-AMS and compared it with that induced by the marketed brimonidine eye drops, i.e., Alphagan P. The results showed that in BMD-AMS, the duration in the decrease in IOP and the area under the drug concentration in the aqueous humor-time curve was found to be 12 h and 2.68 μg/mL, respectively, which is twice as compared to Alphagan P. These finding indicated enhancement in ocular bioavailability of brimonidine with BMD-AMS.

### 4.5. Magnetic Nanoparticles (MNPs)

Magnetic nanoparticles (MNPs) belong to the group of nanotechnology-based materials consisting of magnetic elements such as iron, cobalt, chromium, manganese. It can be used for various biomedical applications such as drug delivery, magnetic resonance imaging, tissue repair, transfection and tissue targeting [95]. It forms a strong drug delivery system as their reactive surface can be functionalized with biocompatible coatings or bioactive molecules, which increase their specificity toward cellular targets and avoid their interaction with healthy cells [96]. In addition to this, MNPs also provides two other advantages; first, it can be controlled by noncontact forces, second, it has potential to be used in MRI tracking and could be used in targeted nano-drug delivery system [96].

Most used MNPs include iron-oxide magnetic particles. The coating of biocompatible material on iron-oxide magnetic particles avoids aggregation, biodegradation, and alterations of particles from their original state and allows entrapping of the bioactive agent on the particle via covalent attachment or adsorption [97]. It has a magnetite or maghemite core surrounded by a shell that contains a layer of polymer or functional group (such as antibodies, biotin, amines and streptavidin) [69]. The drug molecules are attached to the shell of magnetic nanoparticles. The main advantage of using magnetic iron oxide nanoparticles is that they can be visualized by magnetic resonance imaging, and the drug-loaded nanoparticles can be held in place by using a magnetic field. In a study done by Yanai et al. [98], superparamagnetic iron oxide nanoparticles (SPIONs) were used to magnetized rat mesenchymal stem cells (MSCs) in order to deliver cells to the diseased area in the dystrophic retina. Mesenchymal stem cells (MSCs) labeled with fluidMAG-D were injected intravitreally in a retinal degenerative transgenic rat. In vitro studies revealed that cells remain viable and retained differentiation ability even after magnetizing them using fluidMAG-D. The results showed that magnetic MSC delivery to the retina increased tenfold as compared to the normal intravitreal injected cells. Cryosection imaging revealed that magnetic MSC cells had migrated into the inner as well as outer retina. Moreover, magnetic nanoparticle treatment along with orbital magnet resulted in significantly higher concentrations of anti-inflammatory molecule IL-10 and hepatocyte growth factor in the retina. These findings suggested that this approach may provide optimal benefit in outer retinal diseases like AMD where controlled delivery to focal cells is required because it can deliver a higher drug load to the site of interest and resulted in therapeutically useful biochemical changes in the dystrophic retina.

In a study on 44 Sprague–Dawley rats, polymer-coated 50 nm or 4 µm MNPs were injected in the left eye, and the same amount of PBS into the right eye and their effect can be evaluated on IOP, corneal endothelial cell count, the morphology of retina and functioning of photoreceptors at different times [99]. Measurement of IOP, ERG and histology showed no toxicity after injecting magnetic nano- and microparticles. However, microparticles showed small toxicity in corneal endothelial cell counts and iron deposition in tissues. In another study, polymer-coated MNPs were found to be nontoxic to the photoreceptors at histologic as well as electrophysiologic levels [100]. Table 2. Summarizes the applications of inorganic nanoparticles for drug delivery to ocular surfaces.

### 4.6. Implant Devices

Implant devices made from polymeric materials can be used as a mode of drug delivery. They showed controllable release of the drug over a long period of time [69]. Based on the material, implants can be classified into two types; biodegradable and non-biodegradable devices. Non-biodegradable implants have shown more accurate control of drug release and longer release periods as compared to biodegradable implants. However, surgical removal of non-biodegradable devices may cause problems for a patient. Based on the places of implantation, the release of drugs in the retina may range from five weeks to six months. Currently, there are several types of commercial implants that are available for ocular disease treatment, such as Ozurdex (dexamethasone biodegradable implant; Allergan, Inc., Irvine, CA, USA), Trivaris (triamcinolone acetonide suspension; Allergan, Inc.), Kenalog (triamcinolone acetonide suspension; Bristol-Myers Squibb, Princeton, NJ, USA), Triesence (triamcinolone acetonide suspension; Alcon, Fort Worth, TX, USA), Retisert (fluocinolone acetonide non-biodegradable implant; Bausch and Lomb, Inc., Rochester, NY, USA), Iluvien (fluocinolone acetonide non-biodegradable implant; Alimera Sciences, Inc., Alpharetta, GA, USA) [69].

## 5. Potential Ocular Nanomedicine in Clinical Trials and on the Market

The astounding potential of nanomedicine in the pharmaceutical field to improve health care has captured scientists’ attention, with extensive research globally to acquire a market position [101,102]. The first product of nanomedicine was placed on the market almost two decades ago. As of 2012, 33 nanotherapies were available on the market with more than 100 therapeutics in development, and around 50% of nanomedicine companies were based in the United States [103,104]. Numerous nanomedicine products have been under investigation in clinical trials, and some have been approved by the US Food and Drug Administration (FDA) and are available to use in clinical settings for the treatment of conditions such as cancer, autoimmune disease, infectious disease, age-related macular degeneration and others [105]. The great evolution of nanotechnology has dominated the drug delivery field, leading to the progression of formulations for ocular drug delivery, with many in clinical trials and some already introduced in the market.

Many nanoformulations for ocular therapies have been developed and commercialized in the market, but some formulations such as drug-loading emulsions are still slow in progressing for therapy even though drug-free nanoemulsion has been approved to treat dry eye. Restasis^®^ is the first product on the market developed as a nanoemulsion containing cyclosporin A for chronic dry eye treatment, and Durezol^®^, a nanoemulsion containing the drug difluprednate, is approved to treat inflammation of the eye [7]. Another ocular nanomedicine is Visudyne^®^, a liposomal formulation containing verteporfin marketed by Novartis Pharma Ag that was approved by the FDA in 2000 for intravenous administration in the treatment of choroidal neovascularization due to age-related macular generation, pathological myopia, and ocular histoplasmosis syndrome. Macugen^®^ is a PEG anti-vascular endothelial growth factor aptamer administered by intravitreal injection that was approved by the FDA in 2004 for wet age-related macular degeneration treatment [106]. According to Grumezescu, many ophthalmic nanoformulations are under development in clinical evaluation, including TLC399 (ProDex), which contains dexamethasone sodium phosphate and is currently in phase II for treatment of macular edema due to retinal vein occlusion. In addition, latanoprost coated liposome (POLAT-001) has completed phase II clinical trials for ocular hypertension and primary open-angle glaucoma treatment [107]. Based on clinicaltrials.gov, a website developed by the U.S. National Institutes of Health, Department of Health and Human Services, one current ocular nanomedicine under clinical trial in 2020 is SYSTANE^®^, a propylene glycol-based eye drops nanoemulsion, which has completed phase IV for dry eye disease treatment. The development of nanotechnology in ophthalmic therapies seems to be a promising strategy with an increasing number of nano-based formulations in clinical trials and on the market, although more research and studies are necessary regarding nanostructure delivery to the eye [108]. Table 3. Nanomedicine for ocular diseases under clinical trial and approved by FDA.

## 6. Conclusions

The field of nanotechnology has boomed in recent years, accounting for half of the patent filings and one-third of all publications in the US alone. The nanotechnology market has a forecasted compound annual growth rate (CGAR) of 22%, making it a sector with significant investment potential. Around 200 companies and several potential startups have been identified as active in nanomedicine development worldwide. Nanomedicines accounts for approximately 5% of research publications in the field of nanotechnology worldwide. Several nanoparticles, either organic or inorganic, have provided tools that can overcome the problems associated with the conventional drug delivery of ophthalmic formulations. The nanomedicine market is in the early stages of development, and several drug delivery devices are in the clinical testing stage. Various Intravitreal, polymeric drug delivery implants for ocular diseases have been approved by the FDA.

The application of nanotech has shown great potential in ocular nanomedicine research. The nanomaterials could also be used in increasing the bioavailability of various therapeutic agents. Examples of such nanomaterials include liposomes, dendrimers, niosomes, metal NPs. Nanomedicine also has started to showcase its applied potential in improving the pharmacokinetic properties of drug delivery systems; many studies are focusing on integrating hybrid systems with hydrogel using micelles, dendrimers, and cyclo-dextrins [109]. Other aspects that are associated with ocular nanomedicine development have made uniform NPs with reproducible features on an enormous scale. To stimulate clinical translations of nanomedicine, it is critical to investigate the safety and toxicity profiles of such nanomedicine formulations. There could be many other factors that can influence toxicity in nanomedicine, such as administration dose, shape, and size of the particle, surface charge, and functional groups.

Advanced nanofabrication technologies like particle replication in non-wetting templates (PRINT) [110] and the hydrogel template method [111] have been introduced to create ocular nanomedicine. PRINT technology can be used to create monodispersed NPs and microparticles with controlled shape, size, and surface modification at a large-scale. Another applied example that demonstrated the use of PRINT technology is the AR13503 implant, which was manufactured to provide sustained release of API for more than two months in vitro [112]. This implant was prepared using biodegradable polymers PLGA/PDLA/poly(ester amide) (PEA) with rod-shaped and size suitable for injection through a 27 gauge needle. Clinical trials for AR 13,503 are underway and are expected to be used for treating diabetic macular edema (DME) and wet AMD. The hydrogel template method is another advanced nanofabrication technology that can be used to fabricate large amounts of homogenous nanoparticles and microparticles [111]. For example, nanowafer, an ultra-thin transparent lens prepared by filling drug solutions into the dissoluble PVA template. The amount of the drug can be controlled by adjusting the size of the particle.

With several advantages like drug targeting, sustainability, and increased bioavailability, nanomedicine promises to revolutionize the medical markets. Considering the numerous benefits of nanomedicine in ocular drug delivery, aggregation, toxicity, and clearance of nanoparticles remain a concern. Further study of nanodrug carrier development is necessary to provide a more inside understanding of the safety issue and progress in ocular drug delivery.

## Figures and Tables

**Table 1:** Applications of organic nanoparticles for ophthalmic drug delivery.

Nanomaterials	Drug	Application	Animal Model	Function	Ref.
Liposomes	Acyclovir	Topical	Rabbit	Prolong drug penetration	[11]
	Edaravone	Topical	Rabbit	Decrease progression of dry AMD	[12]
	Latanoprost	Subconjunctival injection	Mice	Sustained drug release Reduce intraocular pressure	[13]
Niosomes	Doxycycline hyclate		Rabbit	Prolong drug release rate	[21]
	Tacrolimus	Topical	Rabbit	Increased precorneal drug retention	[23]
Solid lipid nanoparticle	Cyclosporine A	Topical	Sheep	Prolonged drug release	[29]
	Tobramycin	Topical	Rabbit	Increased drug retention	[30]
Polymeric NPs (Eudragit^®^)	Amikacin	Topical	Rabbit	Improved ocular penetration Controlled release	[36]
Polymeric NPs (chitosan)	Cerium oxide		Mice	Improved corneal penetration	[35]
Dendrimers	Acetazolamide	Topical	Rabbit	Enhanced drug residence time	[44]
	Puerarin		Rabbit	Improved corneal permeation	[45]
	Anti-VEGF			Reduced CNV progression	[46]
Nanosuspension	IBU sodium salt	Topical	Rabbit	Increased penetration Prolonged drug release	[50]
	Glucocorticoid			Prolonged drug absorption	[51]
Nanoemulsion	Terbinafine hydrochloride			Improved drug residence time Increased bioavailability	[58]
	Acyclovir			Increased corneal permeation	[59]

**Table 2:** Applications of inorganic nanoparticles for drug delivery to ocular surfaces.

Inorganic Nanomaterial	Drug	Application	Animal Model	Function	Ref.
AuNPs	Resveratrol	Injection		Reduced retinal inflammation	[65]
		Topical	Rat	Decrease the intraocular oxidative damage	[66]
	Immunoglobulin-G	Subretinal injection	Rabbit	Improved biodistribution	[67]
	DNA			Treat retinal dystrophies	[68]
AgNPs			Rat	Produced antioxidants Exhibit anticataractogenic	[75]
				Anti-vasopermeability	[76]
				Antifungal activity	[79]
Cerium oxide NPs			Rat	Reduced photoreceptor damaged	[88]
			Mice	Decreased reactive oxygen species	[85]
		Intravenous injection	Rat	Decrease neurodegenerative	[89]
		Intravitreal injection	Mice	Inhibit neovascularization	[90]
MSNs	Brimonidine	Topical	Rabbit	Enhancement in ocular bioavailability	[91]
MNPs	Mesenchymal stem cells (MSCs)	Intravitreal injection	Rat	Increase tenfold in delivery of drug load to the site of interest	[98]
	Polymer-coated MNPs	Intravitreal injection	Rat	polymer-coated MNPs found to be nontoxic to the photoreceptors	[99]

**Table 3:** Nanomedicine for ocular diseases under clinical trial and approved by FDA.

Product	Nanoformulation	Indication	FDA Approval Status	Ref.
Restasis^®^	Nanoemulsion	Dry eye	Approved	[7]
Durezol^®^	Nanoemulsion	Eye inflammation	Approved	[7]
Ozurdex	Dexamethasone biodegradable implant	Macular edema, Non-infectious uveitis	Approved	[69]
Trivaris	Triamcinolone acetonide suspension	Uveitis	Approved	
Kenalog	Triamcinolone acetonide suspension	Macular edema	Approved	
Retisert	Fluocinolone acetonide non-biodegradable implant	Non-infectious uveitis	Approved	
Iluvien	Fluocinolone acetonide nonbiodegradable implant	Diabetic macular edema	Approved	
Triesence	Triamcinolone acetonide suspension	Macular edema	Approved	
Visudyne^®^	Liposome	AMD	Approved	[106]
Macugen^®^	Aptamer–polymer nanoparticle	Wet AMD	Approved	[106]
TLC399 (ProDex)	Lipid-based nanoparticle	Macular edema	Phase II	[107]
POLAT-001	Liposome	Glaucoma	Phase II	[107]
SYSTANE^®^	Propylene glycol-based nanoemulsion	Dry eye	Phase IV	[108]

## Data Availability

Not applicable.
